# Effect of cations on aerobic granulation for sidestream treatment

**DOI:** 10.1016/j.heliyon.2024.e37216

**Published:** 2024-08-31

**Authors:** Eunyoung Lee, Kyung Jin Min, Ah Hyun Lee, Ki Young Park

**Affiliations:** a Department of Civil, Environmental and Plant Engineering, Konkuk University, 120 Neungdong-ro, Gwangjin-gu, Seoul, 05029, South Korea; b Department of Tech Center for Research Facilities, Konkuk University, 120 Neungdong-ro, Gwangjin-gu, Seoul, 05029, South Korea

**Keywords:** Aerobic granule, Cation, Extracellular polymeric substance, Biomass adhesion, Relative hydrophobicity

## Abstract

Aerobic granular sludge (AGS) represents an aggregate of sludge formed through the self-immobilization of microorganisms under aerobic conditions. It is currently under scrutiny for its potential as a technology to reduce carbon emissions and promote sustainability. The practicality of AGS stems from its ability to encourage granule formation and enhance structural stability. In this study, a total of five cations (K^+^, Ca^2+^, Mg^2+^, Al^3+^, Fe^3+^) were introduced to facilitate stable structuring and the formation of granules for treating high-strength wastewater, such as side-stream treatment. As a result of the experiment, the loosely bound extracellular polymeric substances (LB-EPS) content in the cation-enhanced sludge witnessed a significant increase, leading to elevated total EPS content under all experimental conditions. Furthermore, the protein (PN)/polysaccharide (PS) ratio, a pivotal component of EPS influencing AGS's hydrophobicity and structural stability, exhibited a collective increase, with Mg^2+^ reaching the highest value of 1.7. The relationship between relative hydrophobicity and the PN/PS ratio was found to strongly impact sludge adhesion, with noteworthy results observed particularly for Mg^2+^, Al^3+^, and Fe^3+^. The viability of attached cells reached 96.8 %, the highest recorded in the case of Mg^2+^. In the context of treating high-strength wastewater, Mg^2+^ emerged as the optimal cation for accelerating AGS formation and enhancing structural stability.

## 1 Introduction

The side-stream generated by the anaerobic digestion process in sewage treatment plants contains significant concentrations of organic matter and nutrients. These components contribute up to 25 % of the total nitrogen load in the sewage treatment plant. This situation can result in heightened energy consumption and a decline in the quality of effluent water [1]. As a result, side-stream treatment becomes necessary to mitigate the reintroduction of pollutants into the mainstream.

The aerobic granular sludge (AGS) process, involving the colonization of microorganisms, stands as a promising technology for biological wastewater treatment due to its impressive resistance, settleability, and its ability to concurrently eliminate various contaminants including carbon, nitrogen, and phosphorus [2]. Recent studies indicate that AGS offers the advantage of reducing operational costs and energy consumption in comparison to conventional activated sludge systems, while also minimizing its environmental footprint [3,4]. AGS constitutes a self-immobilized, compact aggregate primarily composed of bacteria and extracellular polymeric substances (EPS). It is characterized by a layered microbial structure that encompasses an outer aerobic layer housing a blend of heterotrophic and autotrophic organisms, along with an anoxic or anaerobic core harboring denitrifying and anaerobic organisms [5].

However advantageous the wastewater treatment process employing AGS might be, the prolonged formation period and the instability of granule structure are recognized as significant challenges that hinder the industrial application of this technology [6]. Given that AGS's core is comprised of microorganisms and EPS, operational factors such as microbial degradation and mass transfer resistance contribute to the dissolution of the core and eventual granule breakage [5]. Consequently, numerous research efforts are underway to enhance the rate of AGS formation while simultaneously fortifying the stability of its structure. The formation of AGS is influenced by several factors, including seed sludge, substrate characteristics, organic loading rate, pH, temperature, and reactor operating conditions [7]. Among these factors, cations have been identified as potential agents to address the issue of granule breakage due to core decomposition [8]. Cations facilitate increased adhesion between cells and promote the production of EPS, a key factor in aggregating microorganisms. This results in a shortened granule formation period. Additionally, cations can neutralize the negative charge on microbial cells through electrostatic interactions, fostering the formation of microbial aggregates. This, in turn, replaces the role of the core and enhances the structural stability of the granules. Also, the addition of cations can be an easy and controllable method to reduce the long formation time of AGS while minimizing changes to the operating conditions of existing wastewater treatment process. Among the various cations, the addition of calcium has been found to expedite the sludge granulation process [9], while magnesium not only accelerates granulation but also boosts microbial diversity [10]. Iron exhibits the ability to bind with EPS components, thereby encouraging microbial aggregation [11].

An approach to assess AGS formation involves examining the relative hydrophobicity of aerobic sludge alongside EPS analysis [12]. More recently, recognizing the significance of microorganism attachment in AGS formation, a technique for evaluating sludge adhesion has been introduced [13].

While cations have been shown to promote AGS formation, there are currently no reported instances involving high-strength wastewater with elevated levels of organic matter, nutrients, and diverse metal ions, such as side-stream scenarios. Furthermore, research regarding the specific contributions of cations with varying electron valences to AGS formation remains limited. Considering these gaps, this study aims to promote the formation of AGS for side-stream treatment by introducing cations to activated sludge and investigating their individual impacts on AGS formation by changes in the characteristics of the existing activated sludge. To achieve this, five cations (K^+^, Ca^2+^, Mg^2+^, Al^3+^, Fe^3+^), primarily found in actual wastewater treatment plants, were added to the activated sludge to assess their influence on EPS content and composition, relative hydrophobicity, and sludge adhesion. The goal is to identify the optimal cation for AGS formation, capable of yielding a shortened formation period and a stable structure. This investigation seeks to uncover cations that could offer a promising alternative for the treatment of high-strength wastewater.

## 2 Materials and methods

### 2.1 Influent characteristics and batch reactor operation

**Fig. 1:**
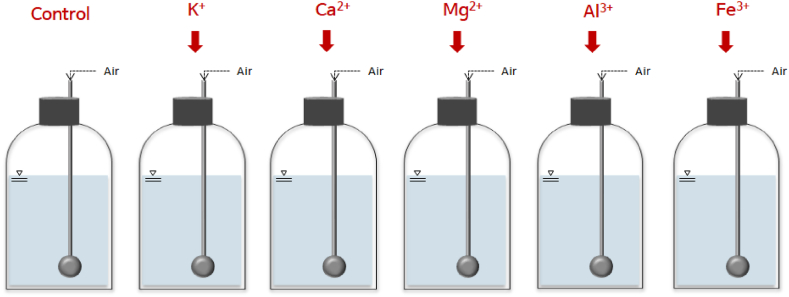
Schematic diagram of batch experiment.

To investigate the effect of cations on the initial formation of AGS, a batch experiment was conducted. In this experiment, 400 mL of activated sludge and an equal amount of synthetic wastewater, designed to simulate side-stream conditions, were combined within a 1 L reactor (Fig. 1). Activated sludge from the Jungnang Water Regeneration Center in Seoul, Korea, which treats domestic sewage, was used, with COD 10,020 mg L^−1^, MLSS 10,280 mg L^−1^ and MLVSS 7160 mg L^−1^. The concentration of synthetic wastewater was prepared as COD 1720 mg L^−1^ (sodium acetate, Samchun Pure Chemical Co., Ltd., Korea), T-N 1060 mg L^−1^ (NH_4_Cl, Samchun Pure Chemical Co., Ltd., Korea), T-P 217 mg L^−1^ (H_3_PO_4_, Samchun Pure Chemical Co., Ltd., Korea). One of the total six reactors was used as a control without adding cations, and K^+^ (KCl, Showa Chemical Co., Ltd., Japan), Ca^2+^ (CaCl_2_·2H_2_O, Samchun Pure Chemical Co., Ltd., Korea), Mg^2+^ (MgSO_4_·7H_2_O, Duksan Pure Chemicals Co., Ltd., Korea), Al^3+^ (Al_2_(SO_4_)_3_·18H_2_O, Samchun Pure Chemical Co., Ltd., Korea), and Fe^3+^ (FeCl_3_·4H_2_O, Junsei Chemical Co., Ltd., Japan) were additionally added to the concentration of 100 mg L^−1^ at each reactor, following the existing literature [14,15] to monitor changes in a short period. To ensure proper mixing, the reactor was stirred at 100 rpm using an agitator, and the temperature was controlled at 25 ± 2 °C. Maintaining aerobic conditions, the dissolved oxygen (DO) concentration was regulated to be at least 3 mg L^−1^. All batch experiments were performed in triplicate.

### 2.2 Analytical methods

#### 2.2.1 EPS extraction and analysis

The extraction of EPS from the sludge was accomplished through the thermal extraction method, as previously outlined in studies by Jang et al. [16]. A 25 mL sample was subjected to centrifugation at 4000 g for 5 min at 4 °C. The resulting supernatant was discarded, and the residual solids were mixed with a phosphate-buffered saline (PBS) solution (0.9 % NaCl) at 70 °C, in a 25 mL volume, employing a vortex mixer for 1 min. Subsequently, another round of centrifugation at 4000 g for 10 min at 4 °C was performed, and LB-EPS was quantified from the supernatant filtered through a 0.45 μm filter paper. Following the discarding of the supernatant, the remaining solids were again combined with the sample in a 25 mL volume of PBS solution (maintained above 50 °C) and subjected to heating in a 60 °C water bath for 30 min. This was followed by centrifugation at 4000 g for 15 min at 4 °C, and TB-EPS was measured in the supernatant filtered through a 0.45 μm filter paper.

Since EPS primarily comprises polysaccharides (PS) and proteins (PN), the EPS content was quantified by summing these two components. Polysaccharides were evaluated using the Bradford method and the phenol-sulfuric acid method, with a standard curve established using glucose as a reference. Protein content was determined through the Lowry method using the Bio-Rad DC protein assay, with a standard curve constructed using the BSA standard [17].

To further understand the impact of cations on the composition of organic materials, both LB-EPS and TB-EPS were subjected to analysis using fluorescence excitation-emission matrix (F-EEM) with an RF-5301 spectrofluorometer (Shimadzu Co., Japan). The fluorescence characteristics of organic materials were examined using an arc lamp, with excitation wavelengths spanning 220–400 nm (at 10 nm intervals) and emission wavelengths spanning 280–600 nm (at 1 nm intervals).

#### 2.2.2 Measurement of relative hydrophobicity

The relative hydrophobicity (RH) of the sludge was assessed using a modified version of the approach detailed by Hao et al. [12]. Following the completion of the batch experiment, a mixture of 5 mL of the sample and 5 mL of n-hexane was prepared. After allowing the two phases to separate completely over a 30 min duration, the aqueous phase was extracted, and its absorbance was measured at 600 nm. The sludge's hydrophobicity was computed utilizing the following Eq. (1):

(1) $$\text{RH}\left(\%\right)=\left(1-A_{0}/A\right)\times 100$$

Here, A_0_ represents the OD_600_ prior to n-hexane treatment, and A corresponds to the OD_600_ post n-hexane treatment.

#### 2.2.3 Biofilm analysis

A biofilm analysis was conducted to evaluate and quantify the formation and characteristics of biofilms influenced by each cation, following the methodology by Song et al. [18]. For this purpose, a piece of polyvinylidene fluoride (PVDF) membrane, measuring 1.5 cm × 1.5 cm, was affixed to a batch reactor, and agitated at 150 rpm for a duration of 24 h within the sludge suspension. Membrane segments hosting attached microorganisms underwent two rinses with a 0.9 % NaCl solution before being subjected to staining utilizing the LIVE/DEAD BacLight Bacterial Viability Kit (Molecular Probe, Eugene, Oregon, USA). This staining agent comprises SYTO 9 and propidium iodide (PI). Specifically, PI, functioning as a red fluorescent dye that penetrates solely into deceased cells with compromised cell membranes, binds to the DNA of dying or lifeless cells, thus emitting intense red fluorescence. Conversely, SYTO 9, a green fluorescent dye, uniformly emits green fluorescence within living cells. To undertake biofilm staining, a mixture consisting of 1.5 μL of SYTO 9 and 1.5 μL of PI solution, combined with 1 mL of distilled water, was prepared. Subsequently, 200 μL of this mixture was pipetted to cover the entire membrane surface. The membrane was then enveloped in aluminum foil, allowing it to be stained in the dark for a period of 30 min. Following staining, careful rinsing with distilled water was performed to eliminate any excessive staining. The stained membrane was subsequently subjected to examination using confocal laser scanning microscopy (CLSM) utilizing an LSM 810 system (Carl Zeiss, Germany). To facilitate this, a cover glass measuring 24 × 60 mm with a thickness of 0.17 mm was placed over a glass slide. Subsequent quantification of CLSM images depicting the biofilm formed on the membrane's surface was executed through the utilization of COMSTAT, an image analysis software [19].

## 3 Results and discussion

### 3.1 Effects of cations on EPS characteristics

**Fig. 2:**
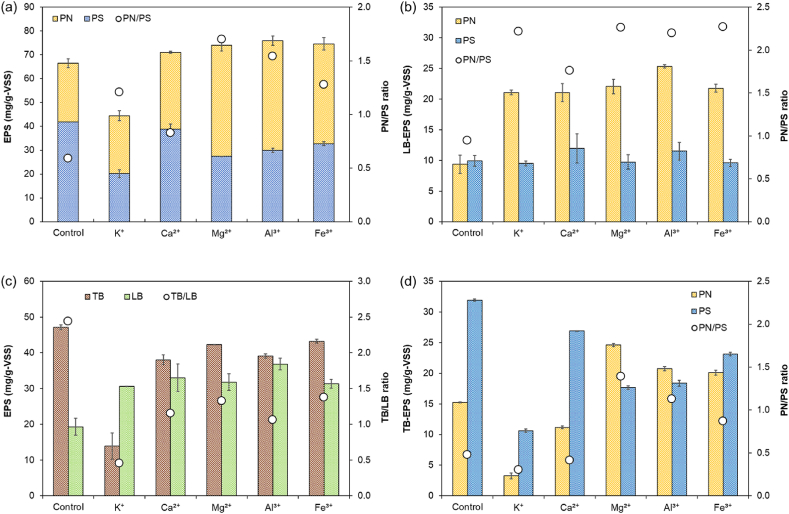
Variation in EPS content in response to cation supplementation: (a) PN and PS of total EPS, (b) PN and PS of LB EPS, (c) TB-EPS and LB-EPS of total EPS, and (d) PN and PS of TB-EPS.

EPS plays a paramount role in microbial aggregation, granulation, and the stability of AGS [20,21]. The alterations in EPS content and composition across all experimental conditions are depicted in Fig. 2. Except for the K^+^ condition, all experimental conditions exhibited an increase in EPS content in comparison to the control (Fig. 2(a)). This augmentation in EPS might be attributed to the bacterial response to the new environment. The enhanced sludge activity within a nutrient-rich milieu could lead to a gradual rise in EPS content. The conditions with heightened EPS content were accompanied by an increase in PN content and a decrease in PS content, in alignment with prior findings [22]. The decline in EPS content upon K^+^ addition could stem from shifts in microbial composition [23] or its utilization as a carbon and energy source in microbial metabolism [24].

Broadly, EPS can be categorized into soluble EPS (S-EPS), which readily dissolves in water and exhibits lower stability under external influences, and bound EPS (B-EPS), which tightly adheres to cells and showcases a dual-layer dynamic structure [[25], [26], [27]]. B-EPS is further subdivided into an inner layer known as tightly bound EPS (TB-EPS), and an outer layer referred to as loosely bound EPS (LB-EPS) [16]. Comprising polysaccharides, organic acids, and other low to medium aromatic compounds, LB-EPS is not conducive to biological flocculation, precipitation, and dehydration [28]. An excess of LB-EPS weakens the adhesion and cohesion of microorganisms, which are crucial in the early stages of AGS formation [29].

As depicted in Fig. 2(b) and (c), LB-EPS increased across all experimental conditions, indicative of an early stage in AGS formation, corroborating prior research outcomes. Moreover, PN exhibited an increase in all experimental conditions, while PS displayed a significant increase only under Ca^2+^ and Al^3+^ conditions. The PN/PS ratio generally fosters the accumulation and aggregation of microbial cells [22]. Given that PS is hydrophilic and PN is hydrophobic, elevating the PN/PS ratio can enhance the relative hydrophobicity of the sludge, consequently bolstering granule stability [30]. The PN/PS ratio of AGS varies greatly, ranging from 0.5 to 16 depending on the experimental conditions [31]. Although the absolute value was lower due to the shorter operation period compared to previous studies, the elevation of the PN/PS ratio within LB-EPS serves as a critical finding indicating that cation addition promotes AGS formation.

However, in contrast, TB-EPS decreased across all conditions (Fig. 2(c) and (d)). Among these, the increase in PN within TB-EPS was most pronounced in the Mg^2+^ condition, registering a 61.2 % augmentation relative to the control. This contrasts with LB-EPS, where the most significant increase was observed in Al^3+^. Unlike Mg^2+^, Al^3+^, and Fe^3+^, K^+^ and Ca^2+^ showed a tendency to decrease PN. This outcome aligns with earlier research asserting that Mg^2+^ is particularly effective in protein synthesis and that Ca^2+^ augments polysaccharide content [32]. The introduction of cations seems to exert a positive influence on AGS formation, as evidenced by the PN increase in LB-EPS. The results of this experiment support previous claims [33] that the PS of TB- EPS might transform into LB- EPS (Fig. 2(d)), though the precise cause remains undetermined. Consequently, further investigation is warranted.

### 3.2 F-EEM fluorescence spectroscopy and EPS transformation

**Fig. 3:**
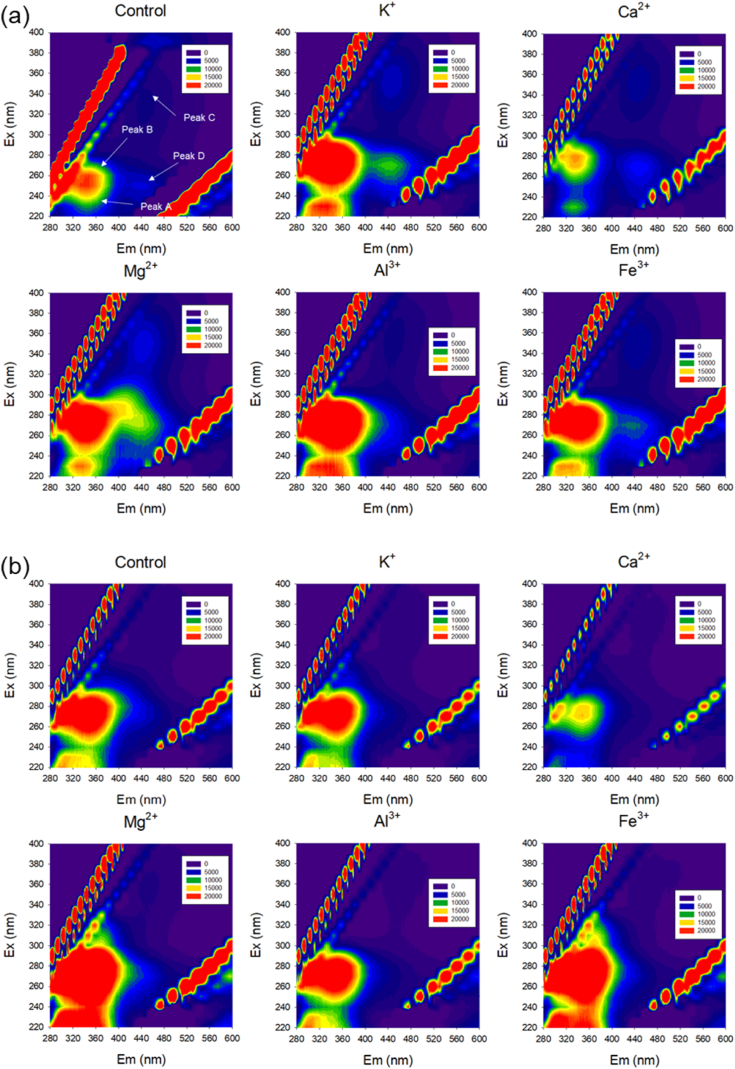
F-EEM fluorescence spectra of (a) LB-EPS and (b) TB-EPS following cation addition.

F-EEM fluorescence spectroscopy emerges as a valuable technique for tracking transformations by discerning distinct fluorescent compounds within complex EPS mixtures (Fig. 3). Each F-EEM spectrum reveals the presence of tryptophan protein-like substances (peak A, Ex/Em = 220–240/330-360), aromatic protein-like compounds (peak B, Ex/Em = 270–280/330-360), humic acid-like components (peak C, Ex/Em = 300–340/400-450), and fulvic acid-like materials (peak D, Ex/Em = 230–260/400-450) within the sludge EPS.

The fluorescence patterns of LB-EPS and TB-EPS exhibit distinct variations in response to different cations. In the case of LB-EPS, protein-like substances corresponding to peak A and peak B exhibit heightened levels compared to the control, with Mg^2+^ inducing the most pronounced increase. Conversely, a decrease is observed with the addition of Ca^2+^. Moreover, the fluorescence intensity of peak C experienced an increase across all experimental conditions. Previous research has indicated that humic substances within EPS could play a significant role in immobilizing exoenzymes through reversible complexation with enzymes [29]. These humic substances in EPS may stem from the degradation of deceased cells and macromolecular organics like PN and PS. Notably, periods characterized by relatively substantial cell lysis, driven by rapid environmental shifts, and heightened endogenous respiration, have been linked to the emergence of humic substances [34]. Thus, these findings strongly suggest that humic substances within LB-EPS are susceptible to environmental fluctuations.

Conversely, for TB-EPS, reductions are observed upon the addition of K^+^, Ca^2+^, and Al^3+^, while increases are evident upon the introduction of Mg^2+^ and Fe^3+^. Additionally, the fluorescence intensity of peak C is notably minimal. It's worth noting that microorganisms exist in two states: planktonic (free-floating) and sessile (attached to surfaces). Microbial attachment to surfaces triggers distinct characteristics between these states, resulting in significant shifts in the expression levels of genes related to EPS production [35].

EPS, constituting 50–90 % of the total organic component, predominantly consists of polysaccharides forming the matrix. The charge of EPS varies, being cationic for gram-positive bacteria such as staphylococci, and neutral or polyanionic for gram-negative bacteria. Ultimately, the chemical composition of EPS can diverge greatly based on the microorganism type [36,37].

Incorporation of carbonyl-containing substituents like hydroxyl, alkoxyl, amino groups, and carboxyl moieties has been reported to lead to red-shifts in peak positions alongside an increase in fluorescence intensity [34]. Consequently, when cations other than Ca^2+^ are present in substantial concentrations, they might be incorporated into LB-EPS or create an environment favoring the dominance of gram-positive bacteria such as staphylococci. In this study, the shifts in peak positions for A, B, and D were more pronounced in LB-EPS than in TB-EPS, suggesting that cations primarily influence LB-EPS due to its proximity to the bulk liquid. The dissimilar peak positions in LB-EPS and TB-EPS due to cations strongly indicate a chemical distinction in EPS composition. Additionally, the minor shift in peak position for TB-EPS might correlate with adhesion. The degree of microbial attachment hinges on a range of factors encompassing material composition, bacterial cell surface properties, temperature, and pressure [38]. Forces governing attachment levels encompass hydrophobic, steric, electrostatic, van der Waals, and protein interactions. As microorganisms encounter and attach to surfaces, loosely connected entities consolidate through an adhesion process driven by EPS production interacting with surface materials and/or receptor-specific ligands present on pili, fimbriae, fibrillae, or the like [35]. Therefore, as previously discussed, the lower fluorescence intensity and slight red-shifts in peak C of TB-EPS could result from microbial consolidation. However, in the context of Mg^2+^, even if microbial solidification occurs, the long-term impact of high-concentration cations on TB-EPS cannot be overlooked.

### 3.3 Relative hydrophobicity and sludge adhesion in response to cations addition

The hydrophobic nature of EPS is well-known, with higher hydrophobicity influencing cell-to-cell interactions and promoting EPS formation [39]. Furthermore, sludge with a comparatively heightened hydrophobicity can facilitate sedimentation by increasing the chances of particle contact, thereby promoting the development of sludge aggregates [12]. Elevated cell surface hydrophobicity encourages cell-to-cell interactions, augmenting the adhesive forces for aggregation. Relative hydrophobicity not only holds significance in microbial adhesion but is also considered a driving factor for granulation.

**Fig. 4:**
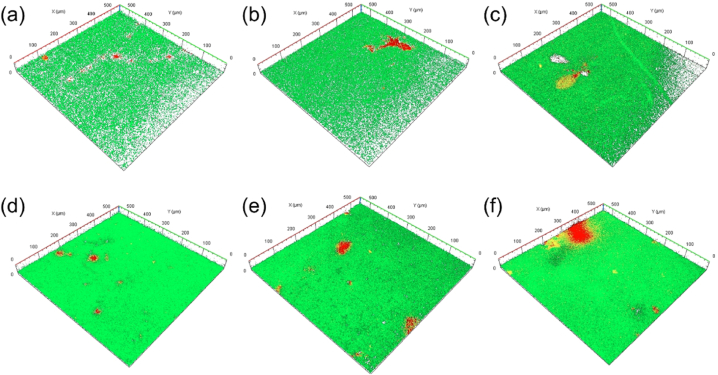
CLSM images of biofilm structure at different cation addition (green: live cell, red: dead cell): (a) control, (b) K^+^, (c) Ca^2+^, (d) Mg^2+^, (e) Al^3+^, and (f) Fe^3^+.

The utilization of confocal laser scanning microscopy (CLSM) image analysis aids in assessing whether cations-induced EPS in microorganisms can effectively bond and aggregate through intercontact between biomass and the hydrophilic membrane. Fig. 4 presents the outcomes of image analysis at the membrane center. The attached biomass exhibits robust metabolic activity across most regions, reflecting vibrant green fluorescence indicative of living cells. While cations enhance adhesion, it is posited that the interaction energy barrier for sludge supplemented with Al^3+^ or Fe^3+^ diminishes considerably compared to Ca^2+^, thereby enhancing the irreversible adhesion of flocs [40]. In this study, cation addition indeed heightens microbial adhesiveness, with Mg^2+^ demonstrating the most pronounced impact on bolstering adhesion, surpassing the effects of Al^3+^ and Fe^3+^. It is inferred that the enhanced microbial aggregation is due to the proteins in the EPS improved by Mg^2+^, rather than the irreversible adhesion resulting from colloid stability and sludge aggregation effects caused by Al^3+^ and Fe^3+^ [40,41].

Table 1 encapsulates the volume of attached biomass and cell viability—indicative of the proportion of live cells among the attached cells—relative to the membrane area. The data in Table 1 and Fig. 4 unveil a direct correlation between the amount of attached and live microorganisms and their corresponding fluorescence. This consistency aligns with the PN/PS ratio of TB-EPS (Fig. 1(d)) and corroborates earlier findings highlighting high TB-EPS content among EPS adhering to membrane surfaces, particularly those rich in PN content [42]. However, the cell viability parameter, which represents the ratio of live cells to cells bearing attached biomass, surpasses 95 % for Mg^2+^ and Al^3+^, yet stands at a mere 87 % for Fe^3+^. This discrepancy might be attributed to the promotion of iron-oxidizing bacteria growth in aerobic environments, consequently generating significant amounts of reactive oxygen species (ROS) through Fenton reactions. This abundance of ROS causes oxidative stress to other microorganisms, potentially leading to cellular damage. Consequently, Mg^2+^ and Al^3+^ appear to exhibit favorable adhesion properties.

**Table 1:** Biofilm properties from COMSTAT analysis.

	Biomass (μm^3^ μm^−2^)		Cell viability (%)
	Live	Dead	
**Control**	0.93	0.12	88.3
**K**^**+**^	6.52	2.21	74.7
**Ca**^**2+**^	7.78	0.53	93.6
**Mg**^**2+**^	11.09	0.37	96.8
**Al**^**3+**^	9.72	0.43	95.8
**Fe**^**3+**^	8.54	1.27	87.0

**Fig. 5:**
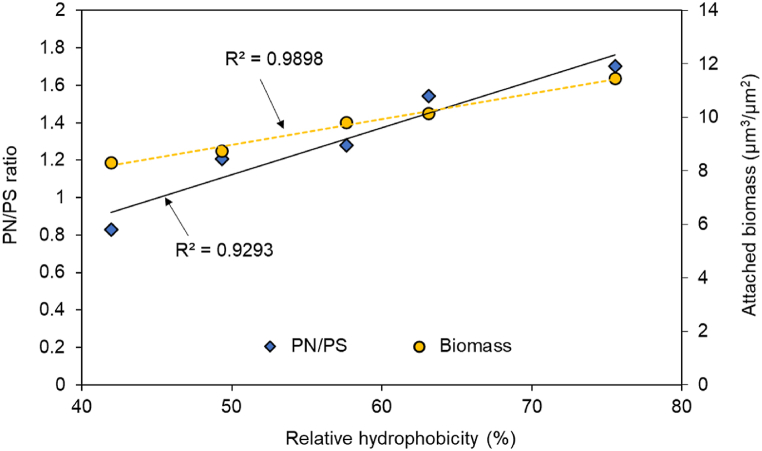
Relationship between relative hydrophobicity and PN/PS ratio, as well as biomass attachment.

The attachment of biomass is influenced by intricate correlations encompassing surface properties, microbial attributes, environmental conditions, and microbe-to-microbe interactions. Among these associations, the connection between relative hydrophobicity, the PN/PS ratio within total EPS, and the biomass attached to the membrane was explored (Fig. 5). Notably, there exists a positive correlation between the relative hydrophobicity of the sludge, the PN/PS ratio, and the biomass quantity. Key contributors to the hydrophobicity of activated sludge are mainly proteins, humic substances, and uronic acids in EPS, while carbohydrates tend to contribute more to hydrophilicity [43]. Therefore, the elevation in extracellular protein content, which enhances relative hydrophobicity, is reasonably inferred to contribute to the promotion of aerobic granulation. This underscores that since the formation of AGS is crucial for augmenting cell binding and interaction, along with EPS secretion, the introduction of cations indeed wields a favorable influence on the AGS formation process.

## 4 Conclusions

In this study, five cations (K^+^, Ca^2+^, Mg^2+^, Al^3+^, Fe^3+^) were introduced into the activated sludge to facilitate the formation of AGS. The assessment encompassed evaluation of EPS content and composition, relative hydrophobicity, and sludge adhesion, with the aim of identifying the optimal cation. Results revealed that, following cation addition, total EPS content increased across all experimental conditions, except when K^+^ was introduced. Notably, LB-EPS exhibited a significant increase, accompanied by a decrease in TB-EPS. The PN/PS ratio in LB-EPS increased notably under all conditions, while in TB-EPS, it increased in the order of Mg^2+^, Al^3+^, and Fe^3+^. A particularly significant rise was observed with the addition of Mg^2+^. Moreover, the addition of Mg^2+^ was associated not only with increased protein content, but also elevated levels of humic and fulvic-like substances. In terms of sludge adhesion, Mg^2+^, Al^3+^, and Fe^3+^ exhibited noteworthy outcomes. The linkage between relative hydrophobicity and the PN/PS ratio was strongly evident in sludge adhesion. The highest viability of attached cells, reaching 96.8 %, was observed upon Mg^2+^ addition. For the treatment of high-strength wastewater, Mg^2+^ emerged as the optimal cation, capable of accelerating AGS formation and enhancing structural stability.

## Data availability statement

Data will be made available on request.

## CRediT authorship contribution statement

**Eunyoung Lee:** Writing – original draft, Visualization, Investigation. **Kyung Jin Min:** Writing – original draft, Validation, Conceptualization. **Ah Hyun Lee:** Methodology, Formal analysis. **Ki Young Park:** Writing – review & editing, Supervision.

## Declaration of competing interest

The authors declare that they have no known competing financial interests or personal relationships that could have appeared to influence the work reported in this paper.

## References

[1] Devos P., Filali A., Grau P., Gillot S. (2023). Sidestream characteristics in water resource recovery facilities: a critical review. Water Res..

[2] Barros A.R.M., Rollemberg S.L. de S., de Carvalho C. de A., Moura I.H.H., Firmino P.I.M., dos Santos A.B. (2020). Effect of calcium addition on the formation and maintenance of aerobic granular sludge (AGS) in simultaneous fill/draw mode sequencing batch reactors (SBRs). J. Environ. Manag..

[3] Ali N.S.A., Muda K., Amin M.F.M., Najib M.Z.M., Ezechi E.H., Darwish M.S.J. (2021). Initialization, enhancement and mechanisms of aerobic granulation in wastewater treatment. Sep. Purif. Technol..

[4] Bengtsson S., de Blois M., Wilén B.M., Gustavsson D. (2019). A comparison of aerobic granular sludge with conventional and compact biological treatment technologies. Environ. Technol..

[5] Franca R.D.G., Pinheiro H.M., van Loosdrecht M.C.M., Lourenço N.D. (2018). Stability of aerobic granules during long-term bioreactor operation. Biotechnol. Adv..

[6] Liu Z., Li N., Gao M., Wang J., Zhang A., Liu Y. (2019). Synergistic strengthening mechanism of hydraulic selection pressure and poly aluminum chloride (PAC) regulation on the aerobic sludge granulation. Sci. Total Environ..

[7] Liu Z., Liu Y., Kuschk P., Wang J., Chen Y., Wang X. (2016). Poly aluminum chloride (PAC) enhanced formation of aerobic granules: coupling process between physicochemical-biochemical effects. Chem. Eng. J..

[8] Ren X., Chen Y., Guo L., She Z., Gao M., Zhao Y., Shao M. (2018). The influence of Fe^2+^, Fe^3+^ and magnet powder (Fe_3_O_4_) on aerobic granulation and their mechanisms. Ecotoxicol. Environ. Saf..

[9] Ren T.T., Liu L., Sheng G.P., Liu X.W., Yu H.Q., Zhang M.C., Zhu J.R. (2008). Calcium spatial distribution in aerobic granules and its effects on granule structure, strength and bioactivity. Water Res..

[10] Li X.M., Liu Q.Q., Yang Q., Guo L., Zeng G.M., Hu J.M., Zheng W. (2009). Enhanced aerobic sludge granulation in sequencing batch reactor by Mg^2+^ augmentation. Bioresour. Technol..

[11] Lin Y., Wang Y., Wang W., Hao T., Su K. (2023). Mechanistic study on the ferric chloride-based rapid cultivation and enhancement of aerobic granular sludge. Environ. Technol..

[12] Hao W., Li Y., Lv J., Chen L., Zhu J. (2016). The biological effect of metal ions on the granulation of aerobic granular activated sludge. J. Environ. Sci..

[13] Zhao T., Qiao K., Wang L., Zhang W., Meng W., Liu F., Gao X., Zhu J. (2021). Isolation and characterization of a strain with high microbial attachment in aerobic granular sludge. J. Environ. Sci..

[14] Lin H., Ma R., Hu Y., Lin J., Sun S., Jiang J., Li T., Liao Q., Luo J. (2020). Reviewing bottlenecks in aerobic granular sludge technology: slow granulation and low granular stability. Environ. Pollut..

[15] Gao D., Liu L., Liang H., Wu W.M. (2010). Aerobic granular sludge: characterization, mechanism of granulation and application to wastewater treatment. Crit. Rev. Biotechnol..

[16] Jang E., Min K.J., Lee E., Choi H., Park K.Y. (2023). Acceleration of aerobic granulation in Sidestream treatment with exogenous autoinducer. Water.

[17] Yang B., Liang W., Bin L., Chen W., Chen X., Li P., Wen S., Huang S., Tang B. (2023). Insights into the life-cycle of aerobic granular sludge in a continuous flow membrane bioreactor by tracing its heterogeneous properties at different stages. Water Res..

[18] Song W., Kim C., Han J., Lee J., Jiang Z., Kweon J. (2023). Application of acyl-homoserine lactones for regulating biofilm characteristics on PAO1 and multi-strains in membrane bioreactor. Membr. Water Treat..

[19] Heydorn A., Nielsen A.T., Hentzer M., Sternberg C., Givskov M., Ersbøll B.K., Molin S. (2000). Quantification of biofilm structures by the novel computer program COMSTAT. Microbiol..

[20] Shen Y., Huang D.M., Chen Y.P., Yan P., Gao X. (2020). New insight into filamentous sludge bulking during wastewater treatment: surface characteristics and thermodynamics. Sci. Total Environ..

[21] Zou J., Yu F., Pan J., Pan B., Wu S., Qian M., Li J. (2021). Rapid start-up of an aerobic granular sludge system for nitrogen and phosphorus removal through seeding chitosan-based sludge aggregates. Sci. Total Environ..

[22] Liu X., Pei Q., Han H., Yin H., Chen M., Guo C., Li J., Qiu H. (2022). Functional analysis of extracellular polymeric substances (EPS) during the granulation of aerobic sludge: relationship among EPS, granulation and nutrients removal. Environ. Res..

[23] Harimawan A., Ting Y.P. (2016). Investigation of extracellular polymeric substances (EPS) properties of P. aeruginosa and B. subtilis and their role in bacterial adhesion. Colloids Surf., B.

[24] Manavi N., Kazemi A.S., Bonakdarpour B. (2017). The development of aerobic granules from conventional activated sludge under anaerobic-aerobic cycles and their adaptation for treatment of dyeing wastewater. Chem. Eng. J..

[25] Dong D., Seo D., Seo S., Lee J.W. (2018). Flocculation of microalgae using extracellular polymeric substances (EPS) extracted from activated sludge. Membr. Water Treat..

[26] Li T., Fan Y., Li H., Ren Z., Kou L., Guo X., Jia H., Wang T., Zhu L. (2021). Excess sludge disintegration by discharge plasma oxidation: efficiency and underlying mechanisms. Sci. Total Environ..

[27] Wang Y., Wang J., Liu Z., Huang X., Fang F., Guo J., Yan P. (2021). Effect of EPS and its forms of aerobic granular sludge on sludge aggregation performance during granulation process based on XDLVO theory. Sci. Total Environ..

[28] Basuvaraj M., Fein J., Liss S.N. (2015). Protein and polysaccharide content of tightly and loosely bound extracellular polymeric substances and the development of a granular activated sludge floc. Water Res..

[29] Li X.Y., Yang S.F. (2007). Influence of loosely bound extracellular polymeric substances (EPS) on the flocculation, sedimentation and dewaterability of activated sludge. Water Res..

[30] Zhang D., Li W., Hou C., Shen J., Jiang X., Sun X., Li J., Han W., Wang L., Liu X. (2017). Aerobic granulation accelerated by biochar for the treatment of refractory wastewater. Chem. Eng. J..

[31] Feng C., Lotti T., Canziani R., Lin Y., Tagliabue C., Malpei F. (2021). Extracellular biopolymers recovered as raw biomaterials from waste granular sludge and potential applications: a critical review. Sci. Total Environ..

[32] Sajjad M., Kim K.S. (2015). Studies on the interactions of Ca^2+^ and Mg^2+^ with EPS and their role in determining the physicochemical characteristics of granular sludges in SBR system. Process Biochem.

[33] Lin H., Zhang M., Wang F., Meng F., Liao B.Q., Hong H., Chen J., Gao W. (2014). A critical review of extracellular polymeric substances (EPSs) in membrane bioreactors: characteristics, roles in membrane fouling and control strategies. J. Membr. Sci..

[34] Zhao L., She Z., Jin C., Yang S., Guo L., Zhao Y., Gao M. (2016). Characteristics of extracellular polymeric substances from sludge and biofilm in a simultaneous nitrification and denitrification system under high salinity stress. Bioprocess Biosyst. Eng..

[35] Sharma S., Mohler J., Mahajan S.D., Schwartz S.A., Bruggemann L., Aalinkeel R. (2023). Microbial biofilm: a review on formation, infection, antibiotic resistance, control measures, and innovative treatment. Microorganisms.

[36] Singh S., Datta S., Narayanan K.B., Rajnish K.N. (2021). Bacterial exo-polysaccharides in biofilms: role in antimicrobial resistance and treatments. J. Genet. Eng. Biotechnol..

[37] Vandana, Das S. (2021). Structural and mechanical characterization of biofilm-associated bacterial polymer in the emulsification of petroleum hydrocarbon. 3 Biotech.

[38] Büttner H., Mack D., Rohde H. (2015). Structural basis of Staphylococcus epidermidis biofilm formation: mechanisms and molecular interactions. Front. Cell. Infect. Microbiol..

[39] Cao F., Bourven I., Van Hullebusch E.D., Pechaud Y., Lens P.N., Guibaud G. (2017). Hydrophobic molecular features of EPS extracted from anaerobic granular sludge treating wastewater from a paper recycling plant. Process Biochem.

[40] Li H., Wen Y., Cao A., Huang J., Zhou Q., Somasundaran P. (2012). The influence of additives (Ca^2+^, Al^3+^, and Fe^3+^) on the interaction energy and loosely bound extracellular polymeric substances (EPS) of activated sludge and their flocculation mechanisms. Bioresour. Technol..

[41] Liu L., Gao D., Zhang M., Fu Y. (2010). Comparison of Ca^2+^ and Mg^2+^ enhancing aerobic granulation in SBR. J. Hazard Mater..

[42] Zhang B., Huang D., Shen Y., Yin W., Gao X., Shi W. (2020). Treatment of municipal wastewater with aerobic granular sludge membrane bioreactor (AGMBR): performance and membrane fouling. J. Cleaner Prod..

[43] Raszka A., Chorvatova M., Wanner J. (2006). The role and significance of extracellular polymers in activated sludge. Part I: literature review. Acta Hydrochim. Hydrobiol..

